# Cerebrovascular Response to Phenylephrine in Traumatic Brain Injury: A Scoping Systematic Review of the Human and Animal Literature

**DOI:** 10.1089/neur.2020.0008

**Published:** 2020-07-23

**Authors:** Logan Froese, Joshua Dian, Alwyn Gomez, Bertram Unger, Frederick A. Zeiler

**Affiliations:** ^1^ Biomedical Engineering, University of Manitoba, Winnipeg, Manitoba, Canada.; ^2^ Department of Surgery, University of Manitoba, Winnipeg, Manitoba, Canada.; ^3^ Department of Human Anatomy and Cell Science, University of Manitoba, Winnipeg, Manitoba, Canada.; ^4^ Department of Internal Medicine, University of Manitoba, Winnipeg, Manitoba, Canada.; ^5^ Center on Aging, University of Manitoba, Winnipeg, Manitoba, Canada.; ^6^ Division of Anesthesia, University of Cambridge, Addenbrooke's Hospital, Cambridge, United Kingdom.

**Keywords:** cerebral blood flow, cerebral blood volume, cerebrovascular reactivity, cerebrovascular response, phenylephrine

## Abstract

Intravenous phenylephrine (PE) is utilized commonly in critical care for cardiovascular support. Its impact on the cerebrovasculature is unclear and its use may have important implications during states of critical neurological illness. The aim of this study was to perform a scoping review of the literature on the cerebrovascular/cerebral blood flow (CBF) effects of PE in traumatic brain injury (TBI), evaluating both animal models and human studies. We searched MEDLINE, BIOSIS, EMBASE, Global Health, SCOPUS, and the Cochrane Library from inception to January 2020. We identified 12 studies with various animal models and 4 studies in humans with varying TBI pathology. There was a trend toward a consistent increase in mean arterial pressure (MAP) by the injection of PE systemically, and by proxy, an increase of the cerebral perfusion pressure (CPP). There was a consistent constriction of cerebral vessels by PE reported in the small number of studies documenting such a response. However, the heterogeneity of the literature on the CBF/cerebral blood volume (CBV) response makes the strength of the conclusions on PE limited. Studies were heterogeneous in design and had significant limitations, with most failing to adjust for confounding factors in cerebrovascular/CBF response. This review highlights the significant knowledge gap on the cerebrovascular/CBF effects of PE administration in TBI, calling for further study on the impact of PE on the cerebrovasculature both *in vivo* and in experimental settings.

## Introduction

l-(3-hydroxyphenyl)-*N*-methylethanolamine or phenylephrine (PE), is an alpha-1 adrenergic drug that is used for systemic blood pressure support in a variety of pathologies. It is one of the most commonly utilized vasopressor agents for cardiovascular support in the management of critically ill patients, through the modulation of adrenergic receptors.^1^ PE is well known as an effective vasopressor that triggers vascular constriction and increases blood pressure.^2^

Emerging methods of biomedical signal processing for recorded cerebral perfusion pressure (CPP) and intracranial pressure (ICP) monitoring have led to targeted patient therapies, with the goal of improving patient outcomes in traumatic brain injury (TBI) based on continuous measures of cerebrovascular reactivity.^3,4^ Vasopressors, including PE, are used to treat patients with critical neurological illness, such as TBI, with the aim of targeting specific mean arterial pressure (MAP) and CPP goals.^1,5^ However, it remains unclear if PE is detrimental to end-organ cerebral perfusion, or leads to impairment of cerebral autoregulation. Such impacts may have important connotations for outcomes in critically ill patients with TBI, as the effective mediation of cerebral blood flow (CBF) or cerebrovascular reactivity may be vital.^6–9^ As such, understanding the impact of exogenously administered PE on cerebrovascular reactivity and CBF is crucial, as individualized care and personalized targets based on cerebral autoregulation and cerebrovascular response are emerging in management strategies for TBI.^8–13^

The goal of this study was to perform a systematic, scoping review of the available literature on the impact of PE on cerebrovascular/CBF response in animal models and human subjects with TBI.

## Methods

A systematic review of the literature was conducted using the methodology outlined in the Cochrane Handbook for Systematic Reviewers.^14^ The data were reported in line with the Preferred Reporting Items for Systematic Reviews and Meta-Analyses (PRISMA).^15^ Supplementary Appendix S1 provides the PRISMA checklist.

The review questions and search strategy were decided on by the supervisor (F.A.Z.) and primary author (L.F.).

### Search question, population, and inclusion/exclusion criteria

The question posed for systematic review was: What is the effect of exogenous systemically administered PE on the cerebrovascular response/CBF in TBI? All studies (animal or human), prospective and retrospective, of any size were included.

The primary outcome measure was the impact on objectively measured CBF or the cerebrovascular responsiveness (i.e., cerebral vasoconstriction or cerebral autoregulation/cerebrovascular reactivity) as documented by: autoradiographic diffusible tracer technique, freely diffusible tracers, thermal diffusion probe, clearance method, laser-Doppler flow probe, flow transducer, flow meter, visual recording software, or any other objective means of CBF determination (including imaging-based techniques). Secondary outcomes included adverse effects of PE administration, including systemic or cerebral end-organ complications.

All studies whether prospective or retrospective, of all sizes, of any age category, and with the use of PE with formal documentation of cerebrovascular response/CBF during administration in the setting of TBI, were eligible for inclusion in this review. Exclusion criteria were: a non-English language study, non-TBI animal model, non-TBI human cohort, or CBF mediation with substance other than PE.

### Search strategy

MEDLINE, BIOSIS, EMBASE, Global Health, SCOPUS, and the Cochrane Library from inception to January 2020 were searched using individualized search strategies for each database. The search strategy for MEDLINE can be reviewed in Supplementary Appendix S2; a similar search strategy was used for the other databases. In addition, the reference lists of reviewed articles on the cerebral blood vessels/CBF response to PE were examined to ensure no references were left out.

### Study selection

Using two reviewers (L.F. and J.D.), a two-step review of all articles returned by our search strategy was performed. First, the reviewers independently screened all titles and abstracts of the returned articles to decide whether they met the inclusion criteria. Second, full text of each returned article was assessed to confirm whether it met the inclusion criteria and that the primary outcome of cerebrovascular/CBF response to PE was documented. Third, the attained literature was separated into TBI versus non-TBI models. Any discrepancies between the two reviewers were resolved by a third party (F.A.Z.).

### Data collection

Data were extracted from the selected articles and stored in multiple electronic databases to ensure data integrity.

### Animal studies

Data abstraction fields included the following: number of animals, type of study, animal model characteristics, the goal of the study, type of vasopressors administered, dose of vasopressors administered, technique of CBF/vasculature assessment, cerebrovascular/CBF response to PE, other outcomes, and general conclusions.

### Human studies

Data fields included the following: number of patients, study type, article location, mean age, patient characteristics, goal of the study, dose, dose duration, technique to measure cerebrovascular/CBF response, the documented cerebrovascular/CBF response, other outcomes, and conclusion.

### Statistical analysis

A meta-analysis was not performed in this study because of the heterogeneity of model types, study designs, and data.

## Results

### Search results and study characteristics

**FIG. 1.**
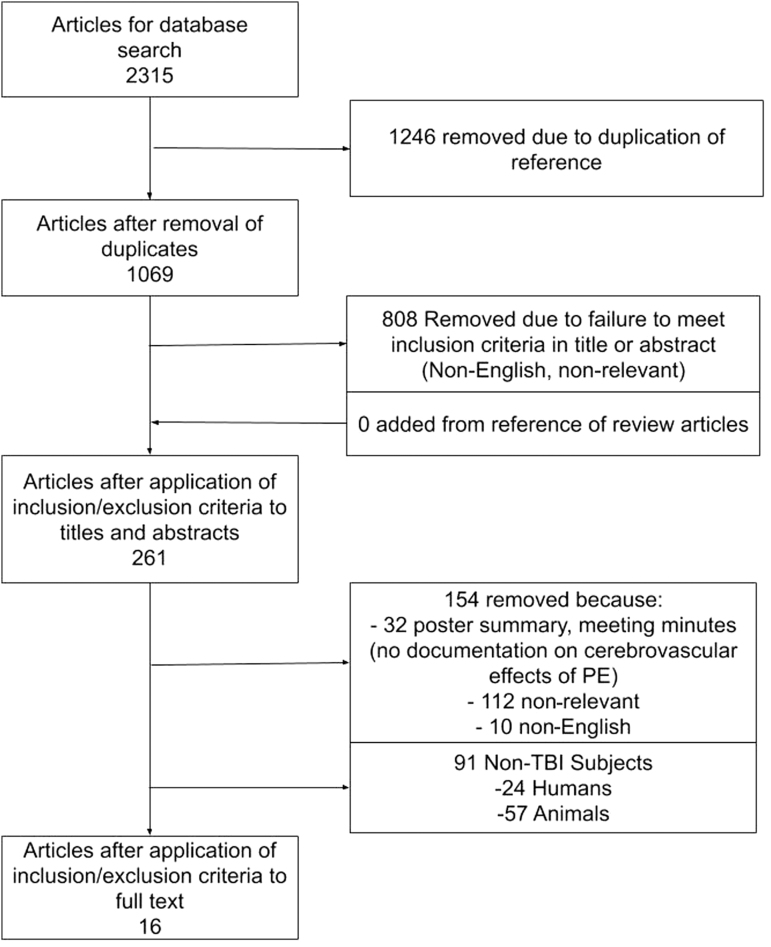
PRISMA (Preferred Reporting in Systematic Reviews and Meta-Analysis) flow diagram of search results and filtering.

The results of the search strategy across all databases and other sources are summarized in Figure 1. Overall, 2315 articles were identified from the databases searched. Removed were 1246 articles because of duplicate references, leaving 1069 to review. By applying the inclusion/exclusion criteria to the title and abstract of these articles, we identified 261 articles that fit these criteria. No articles were added from reference sections of pertinent review articles, leaving a total of 261 articles to review. On applying the inclusion/exclusion criteria to the full-text documents, only 16 articles were found eligible for inclusion in the systematic review, all from the database search. Articles were excluded because they either did not report details around objectively measured cerebrovascular/CBF response to PE administration, were review articles, were non-relevant, or contained non-TBI cohorts.

### Part 1: TBI animal models

Tables 1 and 2 outline the animal TBI study characteristics and cerebrovascular/CBF responses, respectively. Of the 12 articles that had animal models with TBI, the following models were described: induced fluid percussion injury,^16–22^ impact-acceleration injury,^23^ rapid axial head rotation,^24,25^ and rod directed into the parietal cortex.^26,27^ To measure cerebrovascular/CBF response, a variety of techniques were used including: comparing ICP (found with strain gauge or catheter transducer—a surrogate metric for pulsatile cerebral blood volume [CBV]) and partial or region oxygenation (found with near infrared spectroscopy or blood samples), thermal diffusion probes, cortical laser Doppler flowmetry and direct measurement of vasculature change, or change in cerebral tissue weight (i.e., as a metric of CBV). There were 10 studies that used swine models and 2 that used rats.^23,26^ Of the swine model studies, 5 were conducted on newborn pigs,^16–18,24,25^ one had juvenile models,^22^ and the remaining 4 did not specify age (but were assumed to be pre-adult based on weight).^19–21,27^ The majority of studies controlled for both PO_2_ and PCO_2_, while administering heavy sedation.

**Table 1.** TBI Animals Included Studies: General Characteristics and Study Goals

Reference	Number of animals	Study type	Model characteristics	Primary goal of study
Armstead et al.^16^	40 swine	8-arm study	Yorkshire newborn swine anesthetized initially with isoflurane then maintained with fentanyl and midazolam; TBI was induced with a lateral fluid percussion injury. Models had a craniotomy preformed to evaluate vessel change.	Primary: Examination of TBI caused cardiac dysfunction and catecholamine excess and the mediation by a vasoactive agent.
				Secondary: Vasoactive agents' effectiveness to normalize CPP and prevent impairment of cerebral autoregulation.
Feinstein et al.^19^	37 swine	5-arm study	Swine anesthetized with continuous infusions of ketamine, fentanyl, and xylazine; TBI was induced with a fluid percussion injury. Models had a craniotomy preformed to evaluate vessel change.	Primary: Compare initial resuscitation of AVP, PE, or isotonic crystalloid fluid after TBI and vasodilatory shock.
Patel et al.^20^	26 swine	3-arm study	Swine anesthetized with continuous infusions of ketamine, xylazine, and fentanyl with induced TBI and hemorrhage. Models had a craniotomy preformed to evaluate vessel change.	Primary: Evaluate the neurotoxicity, vasoactivity, cardiac toxicity, and inflammatory activity of hemoglobin-based oxygen carrier-201 resuscitation in TBI models.
Dudkiewicz et al.^27^	35 swine	Prospective randomized, blinded animal study	Swine anesthetized with ketamine and xylazine, then ketamine, xylazine, and fentanyl were used to maintain sedation; TBI was induced with blunt force.	Primary: Evaluate tissue oxygenation during management of CPP with PE or AVP.
Malhotra et al.^21^	45 swine	2-arm study	Swine anesthetized with continuous infusions of ketamine, xylazine and fentanyl; TBI was induced with a fluid percussion injury, then animals were bled for 45% of their total blood volume.	Primary: Evaluate the benefits of CPP-directed therapy following TBI models.
Friess et al.^24^	16 piglets	3-arm study	Four-week-old piglets were anesthetized initially with ketamine and xylazine, then maintained with isoflurane; TBI was induced with sagittal head rotations.	Primary: Evaluate PE vs. NE effect on CBF after non-invasive brain trauma.
				Secondary: Evaluate the effects of PE and NE in targeted CPP treatment.
Friess et al.^25^	21 swine	2-arm study	Four-week-old female piglets were anesthetized with ketamine and xylazine, followed by inhaled 4% isoflurane; TBI was induced through a rapid axial head rotation.	Primary: Evaluate PE augmentation of CPP and its subsequent effect on ICP.
Cherian et al.^26^	23 rats	Prospective, randomized study	Male Long-Evans rats, weighing 300–400 g, were fasted overnight and anesthetized with isoflurane; TBI was induced by direct brain tissue impact from a rod.	Primary: Evaluate cerebral hemodynamic effects of PE and L-arginine after cortical impact injury.
Curvello et al^22^	30 swine	6-arm study	Juvenile pigs anesthetized initially with isoflurane, then maintained with midazolam, fentanyl, propofol, dexmedetomidine, and saline; TBI was induced with a fluid percussion injury.	Primary: Evaluate sex and age differences in TBI models with PE treatment of CPP.
Talmor et al.^23^	48 rats	4-arm study	Sprague-Dawley rats were anesthetized with halothane, with TBI induced through closed-head trauma.	Primary: Examine PE-induced hypertension to improve neurological outcomes in TBI models.
Armstead et al.^18^	14 groups of 5 swine	14-arm study	Newborn swine were anesthetized with isoflurane; TBI was induced through a fluid percussion injury.	Primary: Evaluate the potassium channel impairment after TBI in the presence of PE.
				Secondary: The previously stated assessment comparing sex-dependent responses.
Armstead et al.^17^	49 swine	4-arm study	Newborn swine were anesthetized with isoflurane and maintained with a-chloralosed; TBI was induced through fluid percussion injury.	Primary: Evaluate the impairment of cerebral autoregulation during hypotension after TBI through modulation of ERK MAPK.

AVP, vasopressin; CBF, cerebral blood flow; CPP, cerebral perfusion pressure; DA, dopamine; ERK, extracellular signal-regulated kinase; ICP, intracranial pressure; MAPK, mitogen-activated protein kinase; NE, norepinephrine; PE, phenylephrine; TBI, traumatic brain injury.

**Table 2.** TBI Animals Phenylephrine Treatment and Cerebrovascular Response: Study Details

Reference	Dose	Mean administration	Technique to measure**cerebrovascular response	Cerebrovascular response	Adverse effects to phenylephrine	Conclusions
Armstead et al.^16^	PE: 0.8–1.2 μg/kg/min DA: 0.8–1.2 μg/kg/min Papaverine: 10^−8^ and 10^−6^M	Increase CPP to 55–60 mm Hg	Vessel diameter: Closed cranial window technique ICP: Camino transducer	PE in males: Diameter decreased by over 5%, THRR decreased from 1.05 to 0.95(*p* < 0.05) PE in females: Diameter increased by 10%, THRR increased from 1.05 to 1.1 (*p* < 0.05) PE + papaverine: Diameter increased by 15% in both groups DA always increase artery diameter in all groups by up to 25%, THRR increased from 1.05 to 1.20 **PCO_2_ and PO_2_ were not accounted for in vessel changes**	PE and DA reduced ICP in males and females after TBI to roughly the same degree	DA caused a consistent increase to artery diameter and maintained cerebral autoregulation; PE has a varied response that is sex dependent Despite the sex-dependent cerebral vessel response to PE, the increase in CPP indicates an increase in CBF
Feinstein et al.^19^	PE: 0.05–1 mg/kg AVP: 0.1–0.4U/kg	40–300 min	PO_2_: Blood samples SvO_2_: Oximetry ICP: Fiber optic pressure transducer	ICP in all groups decreased by at least 10 mm Hg as compared with the 23 mm Hg of the controls For all groups PO_2_ fell during shock and recovered with resuscitation, which responded to CO_2_ challenges SvO_2_ in solo PE or AVP groups failed to reach 60% but when PE or AVP was given with a crystalloid solution SvO_2_ reached a 60% threshold CPP dropped from 80 mm Hg to 20 mm Hg in all groups during shock, then increased to above 50 mm Hg **PO_2_ and PCO_2_ were controlled**	In addition to vascular hyporesponsiveness in the late stages of vasodilatory shock, catecholamines have significant side effects including tachycardia, arrhythmia, increased myocardial oxygen consumption, and ischemia	To correct vasodilatory shock after TBI, a resuscitation strategy that combined either PE or AVP plus crystalloid solution was superior to either fluid or pressor alone PE and AVP had a non-significant effect on CBF; the ICP/PO_2_ relationship is being used as a surrogate assessment of CBF
Patel et al.^20^	PE: 0.1 mg/mL Mannitol: 1 g/kg	Increase CPP to 70 mm Hg for up to 300 min	ICP: Camino transducer PbtO_2_: NIRS	PE and hemoglobin-based oxygen carrier group had on average about 20 mm Hg higher CPP then control group ICP increased to 30 mm Hg in control group, whereas PE and hemoglobin-based oxygen carrier groups remained at 10 mm Hg PbtO_2_: Returned to baseline value of 100% in PE and control groups, hemoglobin-based oxygen carrier group only reached 50%, and PE and control group responded to CO_2_ challenges; however, hemoglobin-based oxygen carrier group did not **PO_2_and PCO_2_ were controlled**	Not mentioned	PE appears to decrease the overall CBF (using the technique of ICP/SctO_2)_ as compared with control group
Dudkiewicz et al.^27^	AVP: 20 units/mL PE: 10 mg/mL	360 min	ICP: LICOX probe SctO_2_: NIRS	AVP and PE increased CVR, SctO_2_, and ICP AVP: SctO_2_ had 5 mm Hg peaks above baseline during CO_2_ challenges; this increase was also seen within ICP PE: SctO_2_ had 2 mm Hg peaks above baseline during CO_2_ challenges; only after ICP was above 25 mm Hg did the challenges result in peaks PE had an ICP that was higher and lower SctO_2_ than AVP **PO_2_ and PCO_2_ were controlled**	Not mentioned	AVP was as effective as PE for maintaining CPP but at the expense of ICP and SctO_2_ values Cerebrovascular response was found from ICP/ SctO2, which indicates that PE improved cerebrovascular response as compared with AVP
Malhotra et al.^21^	PE: To maintain CPP >80 mm Hg with DA at 2 μg/kg/min	Bolus dose of DA with a continuous infusion of PE	ICP: ICP probe in superior sagittal sinus SvO_2_: Blood samples from catheter	Both PE and control group had a similar rise in ICP after injury to 15 mm Hg SvO_2_ in PE group increased to a baseline of 80%, whereas control group only reached 65% **PO_2_ and PCO_2_ were controlled**	Not mentioned	PE was used to mediate CPP therapy, which saw a significant increase in end SvO_2_. CBF found in the relationship of ICP/SvO_2_ indicates that PE decreased CBF compared with control group
Friess et al.^24^	NE: 7.9 ± 5.2 μg/kg/min PE: 0.9 ± 0.7 μg/kg/min	CPP >70 mm Hg for 5 h	CBF: Thermal diffusion probe ICP: Intraparenchymal monitors	CPP and ICP had similar responses to PE and NE NE resulted in an increase in brain tissue oxygen tension to 25 torr, and PE increased to15 torr from baseline of 10 torr NE increased CBF over time up to 40 mL/100 g/min at 5 h PE increased CBF to 30 mL/100 g/min at 5 h **PO_2_ and PCO_2_ were controlled**	Not mentioned	NE resulted in a greater increase in brain tissue oxygen tension than PE; along with this NE displayed a higher increase in CBF
Friess et al.^25^	PE: Injected to maintain CPP at 40 and 70 mm Hg	6 h	CBF: Thermal diffusion probe PO_2_: Microdialysis probe	Augmentation of CPP to 70 mm Hg by PE significantly improved PO_2_ CBF for the control group was under 10 mL/100 g/min at 6 h, with PE groups having a CBF above 50 mL/100 g/min (*p* < 0.05) **PO_2_ and PCO_2_ were measured, with limited change in PCO_2_**	Not mentioned	The increase in CPP by PE had similar 6-h response in CBF, which was significantly higher than the control group
Cherian et al.^26^	Saline: 1 mL L-arginine: 300 mg/kg/min PE: 0.3 μg/kg/min	Bolus infusion for L-arginine and 4 mL of PE over 3 h	CBF, ICP, and CPP: Laser Doppler flow PO_2_ and :CO_2_: Blood samples	Impact injury increased ICP; and a decreased MAP, CPP, and CBF Saline-treated animals: CBF decreased to 25% of the baseline values at the impact site and stayed at that level for the entire 3-h monitoring period. On the contralateral side, CBF decreased initially and recovered gradually to approximately 50% of the pre-impact baseline value PE and L-arginine increased CBF back to near-baseline levels. PE increased ICP significantly, whereas ICP with L-arginine did not change L-arginine treatment reduced the contusion volume from a median value of 5.28 mm^3^ to 0.63 mm^3^ **PCO_2_ and PO_2_ were measured, with limited change in PCO_2_**	Although the pressor agents are used currently to increase CBF after TBI, other strategies may also increase CBF without the potential adverse effects of induced hypertension	PE increased CBF by increasing CPP L-arginine increased CBF without changing CPP; the improvement in CBF was accompanied by a decrease in neurological injury
Curvello et al.^22^	PE: 0.8–1.3 μg/kg/min	Not mentioned	Pial arterial diameter: Closed cranial window	In both male and female groups PE increased THRR by 0.20 (*p* < 0.05), with a similar increase in arterial diameter (10% for males and females) **PCo_2_ and Po_2_ were measured, with limited change in PCo_2_ and Po_2_**	Not mentioned	PE constricts brain vessels in both male and female models PE preserves cerebral autoregulation after TBI
Talmor et al.^23^	PE: Hypertension increase of 30–35 mm Hg	15 min	CBV and CBF: Tissue was cut and weighed to identify volume; this is correlated with CBF	Control: Tissue injury volume was 335 ± 92 mm^3^ PE: Tissue injury volume was 357 ± 154 mm^3^ (*p* > 0.62) **PCO_2_ and PO_2_ were not accounted for**	The study indicates that post-injury treatment with 15 min of PE-induced hypertension does not attenuate brain edema, reduce tissue injury volume, or improve neurological outcome in rats	PE-induced hypertension increased CPP and blood flow in a rat model of the focal cerebral ischemia
Armstead et al.^18^	PE: 1 mg/kg/min Cromakalim: 10^−8^ to 10^−6^ M CGRP: 10^−8^ to 10^−6^ M	Bolus dose 30 min before and after TBI	Blood pressure, tissue oxygen concentration, and pH: Catheter was inserted into a femoral artery Pial arterial vessel diameter: Measured with a microscope, a camera, a video output screen, and a video microscaler	Cromakalim and CGRP elicited reproducible pial small artery dilation; vasodilation was blunted by TBI PE: Prevented reductions in pial small artery cerebral vasodilation in response to cromakalim and CGRP in females, but further reduced dilation in males PE increased CPP more in females than in males, both after TBI or combined hypotension and TBI **PCO_2_ and PO_2_ were not accounted for**	CPP via PE sex-dependently improves impairment of cerebral autoregulation seen after TBI through modulation of ERK MAPK upregulation, which is aggravated in males, but is blocked in females	Autoregulation of CBF is dependent on intact functioning potassium channels These data suggest a role for sex-dependent mechanisms in the treatment of the impaired cerebral autoregulation seen after pediatric TBI, therefore PE has a tendency to increase CBF in females but result in CBF being unaffected or reduced in males
Armstead et al.^17^	PE: 1 μg/kg/min Papaverine: 10^−8^ and 10^−6^ M	Not mentioned	Pial artery diameter: Cranial window technique for measuring pial artery diameter CBF velocity: Transcranial Doppler ICP: Camino transducer	PE: Reductions in pial artery diameter, CBF, CPP, and elevated ICP after TBI in males compared with females PE in males resulted in CBF decreased by 20 mL/min/100 g PE in females: Decreased impairment of hypotensive pial artery dilation after TBI Papaverine-induced pial artery vasodilation not effected by TBI and PE CBF, CPP, and autoregulation index decreased markedly during hypotension and TBI in males but less in females ERK MAPK was increased more in males than females after TBI PE blunted ERK MAPK upregulation in females, but increased ERK MAPK upregulation in males **PO_2_ and PCO_2_ were controlled**	Not mentioned	Data indicate elevation of CPP with PE sex dependently prevents impairment of cerebral autoregulation during hypotension after TBI through modulation of ERK MAPK In males CBF tends to decrease, whereas in females CBF remains constant

AVP, vasopressin; CBF, cerebral blood flow; CO_2_, carbon dioxide; CPP, cerebral perfusion pressure; CVR, cerebrovascular resistance; DA, dopamine; ERK, extracellular signal-regulated kinase; h, hours; ICP, intracranial pressure; MAPK, mitogen-activated protein kinase; min, minutes; NE, norepinephrine; NIRS, near infrared spectroscopy; PbtO_2_, brain tissue partial oxygen pressure; PCO_2_, partial pressure of carbon dioxide; PE, phenylephrine; PO_2_, partial pressure of oxygen; TBI, traumatic brain injury; THRR, transient hypothermic response ratio; SctO_2_, tissue oxygen saturation; SvO_2_, venous oxygen saturation.

#### CBF/CBV response

A clear and consistent increase in CPP was noted in all studies in which subjects had PE injected systemically.^16,19–22,24–27^ However this change did not always translate to an increase in CBF or ICP (a surrogate measure of CBV), shown by the lack of impact on ICP and CBF in two studies.^21,25^ In the studies in which cerebral autoregulation was impaired, there was a significant increase in ICP and CBF caused by PE injection (cerebral autoregulation was impaired with a fluid percussion injury).^16–18,22^ These studies evaluated the CBF before and after injury, as well as before and after PE injection, with CBF increasing toward^16^ or exceeding baseline values post-injection.^17,18,22^ One study evaluated cerebral autoregulation (direct visual change in pial blood vessels) during PE injections, with cerebral autoregulation becoming impaired in male piglets only.^16^ Further, in the setting of a traumatic impact (rod injected to a deformation of 3 mm in parietal cortex) with sodium nitroprusside infusion, a decrease in CBF and ICP occurred, which was then reversed with PE injection back to near baseline values.^26^ In a study utilizing systemic injection of PE, and that measured brain tissue mass change, there was a direct increase in tissue volume mass (correlated with CBV).^23^

#### Direct effect on cerebral vessels

The studies that measured the direct effect of PE on cerebral vessels were all neonatal models. Similar effects of PE in these studies were shown within cerebral vessels, with all studies that measured cerebrovascular diameter or tension demonstrating cerebral vasoconstriction. In two studies, PE was injected directly through a cranial window on the vessels, demonstrating a reduction in pial artery diameters.^17,22^ One study that used the cranial window technique, but utilized systemic injection of PE, found mixed results to vessel change.^16^ Another study found that pial arterial diameter decreases in male piglets during systemic PE injection, but increases in female piglets.^18^

### Part 2: TBI human models

Tables 3 and 4 outline the human TBI study characteristics and cerebrovascular/CBF responses, respectively. There are four studies with human patients with TBI who were injected with PE, and had CBF or cerebrovascular responses measured.^28–31^ One study did not specify the ages of the patients, who had a Glasgow Coma Scale (GCS) score ≤8.^28^ The other three studies all had patients with mean ages of 30–35 years. Of these three studies, one had patients with a GCS score between 3 and 14,^29^ one had patients with diffuse brain injury and a GCS score ≤12,^30^ and one had patients with a mean GCS score of 5.5 and an injury severity score of 37.^31^ In these studies, CBF was measured with the ^133^Xenon inhalation method,^28,29^ the arterial jugular difference in oxygen,^30^ or computed tomography (CT) perfusion.^31^ Most studies adjusted for PCO_2_ but failed to mention whether PO_2_was controlled. Sedative regiments for these studies were not clearly indicated.

**Table 3.** TBI Human Included Studies: General Characteristics and Study Goals

References	No. patients	Study type	Article location	Mean age	Patient characteristics	Goals of study
Bouma et al.^28^	35 patients	Prospective cohort study	Journal	Not mentioned	Severe head injury with GCS score ≤8 for at least 6 h, with no history of cardiac disease; anesthesia was not discussed	Primary: Identify the relationship between cardiac output and CBF
						Secondary: Compare the previously describe relationship in patients with intact vs. impaired autoregulation
Oertel et al.^29^	Transient hyperventilation: 27 patients PE: 26 patients Propofol: 21 patients	Prospective cohort study	Journal	33 ± 13 years	GGS score of 3–14 underwent a total of 70 vasoreactivity testing sessions from post-injury for 0–13 days; anesthesia was not discussed	Primary: Efficacy of hyperventilation, blood pressure elevation, and metabolic suppression therapy in controlling ICP after head injury
						Secondary: Factors that are predictive of ICP reduction
Sahuquillo et al.^30^	46 patients	Retrospective cohort study	Journal	29.7 ± 11 years	Moderate or Severe head trauma with diffuse brain injury type 2 or 3 and GCS score ≤12; anesthesia was not discussed	Primary: To document false autoregulation in patients with severe head injury
						Secondary: Autoregulation's importance in CPP management
Peterson et al.^31^	Cerebral autoregulation was intact in 25 patients	Retrospective cohort study	Journal	35 years	Severe TBI with GCS score of 5.5 and injury severity score of 37; anesthesia was not discussed	Primary: Use computed tomography perfusion to guide blood pressure manipulation in TBI
	Cerebral autoregulation was disrupted in 8 patients					

CBF, cerebral blood flow; CPP, cerebral perfusion pressure; DA, dopamine; E, epinephrine; GCS, Glasgow Coma Scale; NE, norepinephrine; PE, phenylephrine; TBI, traumatic brain injury.

**Table 4.** TBI Human Phenylephrine Treatment and Cerebrovascular Response: Study Details

References	Dose	Mean duration of dose administration	Technique to measure cerebrovascular response	Cerebrovascular response	Other outcome	Conclusions
Bouma et al.^28^	PE: 80mg/500 mL Arfonad: 500 mg/500 mL Mannitol: 0.66 mg/kg was given only to patients who had not received this drug during the previous 4 h and who were not likely to need it in the next 8 h (ICP <20 mm Hg and stable)	To raise MAP by 30%, usually for 20 min Mannitol was given in a 20% solution for 3 min	CBF: ^133^Xenon inhalation or intravenous injection method Cardiac output: Thermodilution method PCO_2_: Capnometer	PE:Intact autoregulation found a CBF change of -1 ± 12% Defective autoregulation found a CBF change of 53 ± 20% (*p* < 0.05) Arfonad: Intact autoregulation found a CBF change of -2 ± 8% Defective autoregulation found a CBF change of -31 ± 1% Mannitol: Intact autoregulation found a CBF change of 0 ± 12% Defective autoregulation found a CBF change of 40 ± 35% (*p* < 0.05) In all groups ICP did not significantly change **PCO_2_ was maintained at 34 mm Hg; PO_2_ was not accounted for**	Cardiac output varied significantly after injection in all groups	No correlation existed between the changes in cardiac output and the changes in CBF, regardless of the status of blood pressure autoregulation A significant increase in CBF was found after administration of mannitol and PE when autoregulation was defective
Oertel et al.^29^	Propofol: 1 mg/kg PE: 10–100 ug/kg/min	Propofol was administered over 10 min, followed by an infusion of PE increasing every 5 min	CBF: ^133^Xenon assessment and transcranial Doppler ultrasonography recordings of the MCA SjvO_2_: Jugular and venous blood samples	Hyperventilation therapy: Patients experienced a mean decrease in PCO_2_ from 35 ± 5 to 27 ± 5 mm Hg, ICP from 20 ± 11 to 13 ± 8 mm Hg (*p* < 0.001) CBF velocity from 111 ± 45 to 86 ± 37 cm/sec SjvO_2_ from 73 ± 8 to 67 ± 8% (*p* < 0.001) PE: ICP increased from 16 ± 9 to 19 ± 9 mm Hg (*p* = 0.001) CBF velocity from 71 ± 27 to 76 ± 29 cm/sec SjvO_2_ from 72 ± 7 to 74 ± 9% (*p* < 0.001) Propofol: ICP decreased from 20 ± 10 to 16 ± 11 mm Hg (*p* < 0.001) CBF from 39.8 ± 13.8 to 33.5 ± 12.9 mL/100g/min (*p* < 0.001) SjvO_2_ increased from 72 ± 8 to 75 ± 8% **PCO_2_ was measured and found to decrease on average in those who underwent hyperventilation; PO_2_ was not accounted for**	Predictors of an effective reduction in ICP included a high PCO_2_ for hyperventilation, a high study GCS score for induced hypertension, and a high PCO_2_ and a high CBF for metabolic suppression	Of the three modalities tested to reduce ICP, hyperventilation therapy was the most consistently effective CBF velocity was increased as a result of the PE-induced hypertension therapy Metabolic suppression therapy and hyperventilation therapy demonstrated a decrease in CBF velocity
Sahuquillo et al.^30^	PE used to induce MAP increase by 20 mmHg	Not mentioned	ICP: Camino transducer Blood oxygenation: Fiberoptic catheter CBF: Found from arterio-jugular difference of oxygen	CBF: 50.5% of patients had a 20% increase and 13.7% of patients had a 20% decrease; the rest had a non-significant response CPP: Had an average increase of 11.5 mm Hg but significant variation **Patients with constant PCO_2_ were included; PO_2_ was not accounted for**	CPP management of autoregulation is not recommended due to the various responses	Increasing MAP to obtain a better CPP in patients is not beneficial because CBF is not modified or may even be reduced PE demonstrated a variation in CBF and ICP response with limited correlation
Peterson et al.^31^	PE infusion was used to raise the CPP by 20 mmHg	Not mentioned	CBF: CBV/mean transit time from CT scan ICP: Camino transducer	PE resulted in minimal changes to CBF in 75.7% of patients, with 24.3% having a notable diffusion increase The mean ICP change was 3.8 mm Hg for the disrupted autoregulation group and 1.5mm Hg for the intact autoregulation group (*p* < 0.006) **PCO_2_ and PO_2_ were not accounted for**	Using direct measurement of CBF in response to a CPP challenge found autoregulation disruption to be much less common than other reports of similar groups	PE injections increase CPP, which either increases CBF or causes no significant change

AVP, vasopressin; CBF, cerebral blood flow; CBV, cerebral blood volume; CPP, cerebral perfusion pressure; CVR, cerebrovascular resistance; ICP, intracranial pressure; MAP, mean arterial pressure; MCA, middle cerebral artery; PCO_2,_ partial pressure of carbon dioxide; PE, phenylephrine; PO_2_, partial pressure of oxygen; SjvO_2_, jugular venous oxygen saturation.

#### CBF/CBV response

All studies objectively documented the CBF response to PE injection in human patients with TBI. One study demonstrated an increase in CPP, with concordant MAP increase.^30^ The CBF response in this study varied and was inconsistent across the population, demonstrating both increases and decreases. A second study failed to demonstrate a significant change in CBF or ICP (i.e., surrogate of pulsatile CBV).^31^ A third study compared patients with intact versus impaired cerebrovascular reactivity (measured by the percentage change in CPP/CBF with the clearance method) and found that PE substantially increased CBF, by up to 53%, when regulation was impaired versus not impaired.^28^ Regarding the CBV response to PE administration, an effect on ICP was also shown, with those displaying impaired cerebrovascular reactivity having a higher mean change in ICP of 3.8 mm Hg versus 1.5 mm Hg to PE injection, for the impaired versus intact groups, respectively (cerebrovascular reactivity was consider intact if an increase in CPP did not change CBF as measured by CT perfusion).^31^

#### Cerebrovascular response

No human study measured the direct cerebrovascular response to PE in human subjects. Despite this, there were some studies that attempted to quantify cerebrovascular resistance (CVR) from the CPP/CBF relationship.^28,30^ However, there was no consensus on the CVR response to PE administration, with CVR demonstrating a variable response.

### Adverse events to phenylephrine in all models

The adverse effects of PE in animal models included: bradycardia, vascular hyperresponsiveness in the late stages of vasodilatory shock, tachycardia, arrhythmia, increased myocardial oxygen consumption, and ischemia.^19,23^ One study showed an increased expression of cerebral V1 receptor subtype after TBI, and the subsequent formation of cerebral edema.^19^ In two studies, one animal and one human, cerebral autoregulation was impaired with PE administration (i.e., there was an in-phase direct increase in CBF with increased MAP).^17,28^ Impaired autoregulation appeared to demonstrate a sex predominance in another study, which found PE induced impaired autoregulation preferentially in male neonatal pigs, as compared with females.^17^

## Discussion

PE is a commonly used vasopressor agent to increase MAP in a variety of treatment practices. In critical care patients, and specifically those with TBI, PE may be utilized in targeting of ICP, CPP (and hopefully CBF) to improve outcomes. Knowledge of the cerebrovascular response to various commonly utilized vasopressor agents, such as PE, is crucial for the provision of effective treatments in TBI.^4,6–11,13,32,33^ The current literature on the cerebrovascular effects of PE leaves uncertainty in predicting the cerebrovascular/CBF response to its administration. Through our scoping review we attempted to gather all studies that documented the administration of PE and measured its effects on the cerebrovascular response/CBF, in both animal models and human subjects afflicted with TBI. Some important points and preliminary trends can be gleaned from this review, although it must be acknowledged that, overall, the literature in this area is limited, highlighting a significant knowledge gap.

First, PE injection leads to a direct increase in MAP, and subsequently CPP. MAP increase is reported in all studies regardless of design or method, and subsequently led to a CPP increase in all studies that concurrently evaluated ICP. This general finding is not surprising, as the main role for PE in critical neurological illness is the augmentation of MAP and CPP. Particularly, in the treatment of moderate/severe TBI, this aspect of PE is attractive and is employed to maintain ICP/CPP targets, as suggested by current guideline-based therapies.^4,34^ However, the exact dose-response relationship between MAP and CPP was not clearly delineated in many studies, particularly in the human studies. As we transition to personalized physiological targets in TBI care, such as optimal CPP,^10,11,13^ we may find ourselves targeting CPP values well above the current Brain Trauma Foundation defined target range of 60–70 mm Hg.^4^ As such, with increasing PE dosing, the relationship between PE dose, MAP, and subsequently CPP may prove to be non-linear. In fact, with escalating doses of PE and the subsequent increase in MAP, there is the potential to overwhelm cerebrovascular reactivity and cause degeneration of the classic Lassen autoregulatory curve.^35^ This would expose patients to significant secondary injury, and poor long-term outcomes, as reported in the human TBI literature.^7,33,36–38^ The current literature in both animal and human TBI fails to inform whether such an upper limit of PE dosing exists. It is likely the specific dose-response is dependent on a variety of individual factors, including genotype.^39^ Thus, despite the similar results seen with PE administration in both animal and human TBI studies, a true understanding of its impact on the cerebral vasculature remains limited, necessitating future investigation.

Second, there were a limited number of TBI animal model studies that measured directed cerebral vessel response, with only four studies in neonate piglet models.^16–18,22^ The literature lacks homogeneity in model type and study design. However, PE administration demonstrated consistent constriction of the cerebral vessels. It must be acknowledged though that the ability to translate cerebrovascular responses to PE in immature neonatal vessels to mature adult cerebral vessels is limited. Despite the clear limitations of these data, there is sufficient homogeneity in the outcome to suggest that PE has a direct vasoconstrictive effect on the cerebral pre-capillary arterioles. This suggested effect is important; given the strong independent association between impaired cerebrovascular reactivity and poor patient outcome in TBI,^37,38^ it is clear that cerebrovascular reactivity is an important aspect of cerebral physiology that impacts secondary injury exposure and outcome. Administration of vasoactive compounds such as PE may directly impact cerebrovascular reactivity, given cerebrovascular reactivity is believed to occur at the pre-capillary arteriole level.^35,40–42^ As highlighted in this review, literature on an exact dose-response at the pre-capillary arteriole and its impact on continuously measured cerebrovascular reactivity is absent. Therefore the impact of continuous PE dosing on cerebrovascular reactivity in TBI remains unclear, supporting the need for future research.

Third, despite the general increase in CPP, the quantitative CBF response in human models was underinvestigated. Of the two studies that showed a CBF increase with PE injection, either autoregulation was impaired,^28^ or there was a conflicting increase and subsequent decrease in CBF.^30^ The animal literature also demonstrated an increase in CBF. However, in the study design PE was never the sole focus of the study, with other medications co-administered, leading to significant confounding. Without a true understanding of the impact of PE on the pre-capillary vascular response, and subsequently CBF, we remain in the dark regarding safe dosing ranges in TBI.

Finally, within the literature it is unclear what the impact of systemic PE injection is on cerebrovascular reactivity/cerebral autoregulation in animal/human TBI, as there were no studies identified that evaluated the cerebral microvascular change within human models. Thus, it remains unknown if there exists a dose-response effect on cerebral autoregulation, where specific PE dose ranges may lead to impaired autoregulation. The animal literature adds insight to the effects of PE on cerebral vessels as reported in four studies in which PE injected systemically or locally found vessels to constrict.^16–18,22^ Further, one animal study suggested a potential disparity in autoregulatory response to PE based on sex, with males displaying deterioration in cerebral autoregulation caused by PE injection.^17^ However, the lack of direct translation of these data to human models and patient care leaves limitations to be addressed in future studies. Also, there was a lack of consistency in evaluating cerebral autoregulation. Within the human studies alone, there were three different methods used to evaluate if cerebral autoregulation was intact. A refined and accurate way to quantify cerebral autoregulation *in vivo* is needed. Such autoregulatory monitoring should take the form of commonly adopted continuous measures of cerebrovascular reactivity, such as pressure reactivity index (PRx).

Thus, overall, given the outlined trends and limitations of the identified literature in this area, we are left with an inconclusive view of the impact of PE on CBF/cerebrovasculature in TBI. But a “negative” or “inconclusive” systematic review does provide important information regarding the current gap in knowledge. Despite PE being a commonly administered vasoactive compound in neurocritical care, we have a limited idea of its impact on the end organ of our primary interest, the brain. Aside from the general increase in MAP and trend toward an increase in CVR, when measured, the impact on cerebral autoregulation/cerebrovascular reactivity and CBF is unclear. Such a glaring hole in the available TBI literature is both important to identify/quantify—which we believe this review has done—and represents an area where future research is of utmost importance (see “Future directions” section for more details). As such, despite the limited conclusions drawn here, we believe highlighting such limitations/deficiencies will lead to future directed study of the cerebral impacts of PE.

### Limitations

Although this review is comprehensive, there exist significant limitations. First, the overall literature was very heterogeneous in design. This limits how definitive we can be in our statements regarding the impact of PE on CBF, and cerebrovascular response in TBI. Similarly, there was heterogeneity in the co-administration of vasoactive compounds, sedative regimens, and documentation of PCO_2_ and PO_2_ levels during measurement of CBF/cerebrovascular response. Also, the heterogeneity in CBF/cerebrovascular response measurement techniques between studies further limits our ability to consolidate findings between studies. Thus, the conclusions regarding the impact of PE on CBF/cerebrovascular response lack strength. Second, animal models do not necessarily translate directly to human response to PE. Therefore, despite a larger volume of literature on animal TBI models, it remains unknown if any of the data can directly translate to human patients. Similarly, some models focused on young or neonatal animals, in which it is known that the cerebrovasculature is in an immature state. The response of such vessels to PE administration may not reflect the true response in mature animals, or adult humans. As such, we are again limited in our ability to extrapolate results. Third, the disparity in response shown in CBF to PE administration limits our ability to confidently state the impact of PE on CBF, both in animals and humans afflicted with TBI. This is further clouded by the different populations and models studied. Finally, almost all studies identified, both animal and human, failed to focus on the cerebrovascular response of the pre-capillary arterioles to PE administration. The animal studies that did utilized neonatal models. These pre-capillary arterioles, which are believed to be the major players in cerebral autoregulatory response, may behave differently in neonates as compared with adults. Knowledge of the impact of PE on such vessels is crucial, as impaired cerebrovascular function is a known associate of poor outcome in the setting of TBI.

### Future directions

Through this review of the literature on the effect of PE on CBF/cerebral vessel response, we found a trend toward an increase in MAP, CPP, and CVR across all animal models and human subjects with TBI. However, the CBF/cerebrovascular response remains unclear, given the disparity in responses shown. Despite the limited concrete conclusions on the impact of PE on TBI derived, this review has highlighted an important knowledge gap related to a readily administered vasoactive compound in neurocritical care, and we have outlined future avenues that require investigation.

Future work on PE and the cerebrovascular response should focus on the dose response in both animals and humans, evaluating regional disparities in vascular response across the age spectrum and between sex groups, and on the pre-capillary arterioles responsible for cerebral autoregulation. Human TBI research would benefit from multi-center and comprehensive high-frequency physiological data sets. Such data would include accurate and complete treatment markups linked to high-frequency physiology, allowing assessment of the impact of PE administration/dose changes on various aspects of cerebral physiology. The high-frequency physiology data sets would need to comprise full waveform data, allowing for advanced bio-signal analytics, and derivation of continuous measures of cerebrovascular reactivity and compensatory reserve. Similarly, the monitoring modalities employed in this human TBI population would ideally include not only ICP/ICP-derived metrics, but also measures of end-organ CBF (such as regional thermal diffusion probes or multi-channel near infrared diffuse correlation/time-resolved spectroscopy), end-organ oxygen delivery (through brain tissue oxygen probes or near infrared spectroscopy), and cerebral metabolism (through microdialysis). Further, with all monitoring in place, manipulations in PE dosing would need to occur, to ensure a comprehensive understanding of the impact of changes in PE on cerebrovascular physiology *in vivo*.

We envision the experimental animal model work employing both healthy controls and TBI models. An important aspect of such future investigations would be controlling/adjusting for potential confounders. This would require uniform sedation regimens and accurate control of PCO_2_/PO_2_. Evaluation of CBF/cerebrovascular response in such animal models would benefit from continuous high-temporal frequency methods, using clinically applied multi-modal monitoring/signal processing techniques. Such monitoring could, in theory, deploy multiple monitors simultaneously, assessing regional CBF, cerebrovascular reactivity, oxygen delivery (using brain tissue oxygen probes or advance near infrared spectroscopy monitoring), and metabolics (using microdialysis). Within the TBI models, given regional disruption in blood–brain barrier integrity, such high-temporal and spatial continuous monitoring is crucial, as regional disparities in CBF/cerebrovascular response post-TBI may be driven by regional differences in blood–brain barrier functionality. This research should involve the application of multi-modal cerebral physiological monitoring, and the derivation of continuous metrics of cerebrovascular reactivity.

Finally, both human and animal research would benefit from concordant genotyping and assessment of vascular protein/vasoactive small-molecule biomarkers associated with cerebrovascular control.^43^ The outlined multi-faceted, comprehensive approach to monitoring is the only way to improve understanding of the impact of PE administration on CBF/cerebrovascular response in TBI. Such investigations are the ongoing aspirations/work of various European,^44^ North American,^45–50^ and Canadian^51^ collaborative efforts.

## Conclusion

In TBI, the human and animal literature on the cerebrovascular/CBF effects of PE is scarce and significantly limited. Limitations include study and model design heterogeneity and failing to adjust for potential confounders. The identified literature demonstrated a trend toward increased MAP, CPP, and CVR with PE administration. The direct cerebrovascular response to PE administration demonstrated cerebral vasoconstriction in TBI animal models. However, the CBF response to PE administration was heterogeneous, in both animal and human studies identified, with no definitive conclusions. This review highlights a major knowledge gap regarding the impact of PE in TBI. Further research into the CBF and pre-capillary arteriole response to PE administration in TBI is required, as it carries important implications for treatment in moderate/severe TBI.

## Supplementary Material

Supplemental data

Supplemental data

## Abbreviations Used

CBF: cerebral blood flow
CBV: cerebral blood volume
CPP: cerebral perfusion pressure
CT: computed tomography
CVR: cerebrovascular resistance
GCS: Glasgow Coma Scale
ICP: intracranial pressure
MAP: mean arterial pressure
PE: phenylephrine
PRISMA: Preferred Reporting Items for Systematic Reviews and Meta-Analyses
PRx: pressure reactivity index
TBI: traumatic brain injury
