# Supplementary material for: Cerebrovascular Response to Phenylephrine in Traumatic Brain Injury: A Scoping Systematic Review of the Human and Animal Literature
Source: Neurotrauma Rep. 2020 Jul 23;1(1):46–62. doi: 10.1089/neur.2020.0008 (PMC8240891; doi:10.1089/neur.2020.0008)
Supplement: Supplemental data [file Supp_AppendixS2.pdf]

Supplementary Appendix S2. Ovid Search Strategy

1. Phenylephrine.mp. [mp=ti, ab, hw, tn, ot, dm, mf, dv, kw, fx, dq, bt, id, cc, nm, kf, ox, px, rx, an, ui, sy]
2. Metasympatol.mp. [mp=ti, ab, hw, tn, ot, dm, mf, dv, kw, fx, dq, bt, id, cc, nm, kf, ox, px, rx, an, ui, sy]
3. Mezaton.mp. [mp=ti, ab, hw, tn, ot, dm, mf, dv, kw, fx, dq, bt, id, cc, nm, kf, ox, px, rx, an, ui, sy]
4. Neo-Synephrine.mp. [mp=ti, ab, hw, tn, ot, dm, mf, dv, kw, fx, dq, bt, id, cc, nm, kf, ox, px, rx, an, ui, sy]
5. Neosynephrine.mp. [mp=ti, ab, hw, tn, ot, dm, mf, dv, kw, fx, dq, bt, id, cc, nm, kf, ox, px, rx, an, ui, sy]
6. Phenylephrine Hydrochloride.mp. [mp=ti, ab, hw, tn, ot, dm, mf, dv, kw, fx, dq, bt, id, cc, nm, kf, ox, px, rx, an, ui, sy]
7. Phenylephrine Tannate.mp. [mp=ti, ab, hw, tn, ot, dm, mf, dv, kw, fx, dq, bt, id, cc, nm, kf, ox, px, rx, an, ui, sy]
8. 1 or 2 or 3 or 4 or 5 or 6 or 7
9. Cerebrovascular Circulation.mp. [mp=ti, ab, hw, tn, ot, dm, mf, dv, kw, fx, dq, bt, id, cc, nm, kf, ox, px, rx, an, ui, sy]
10. Cerebral Blood Flow.mp. [mp=ti, ab, hw, tn, ot, dm, mf, dv, kw, fx, dq, bt, id, cc, nm, kf, ox, px, rx, an, ui, sy]
11. Cerebral Circulation.mp. [mp=ti, ab, hw, tn, ot, dm, mf, dv, kw, fx, dq, bt, id, cc, nm, kf, ox, px, rx, an, ui, sy]
12. Cerebral Perfusion Pressure.mp. [mp=ti, ab, hw, tn, ot, dm, mf, dv, kw, fx, dq, bt, id, cc, nm, kf, ox, px, rx, an, ui, sy]
13. Circulation, Cerebrovascular.mp. [mp=ti, ab, hw, tn, ot, dm, mf, dv, kw, fx, dq, bt, id, cc, nm, kf, ox, px, rx, an, ui, sy]
14. CBF.mp. [mp=ti, ab, hw, tn, ot, dm, mf, dv, kw, fx, dq, bt, id, cc, nm, kf, ox, px, rx, an, ui, sy]
15. cbfv.mp. [mp=ti, ab, hw, tn, ot, dm, mf, dv, kw, fx, dq, bt, id, cc, nm, kf, ox, px, rx, an, ui, sy]
16. cpp.mp. [mp=ti, ab, hw, tn, ot, dm, mf, dv, kw, fx, dq, bt, id, cc, nm, kf, ox, px, rx, an, ui, sy]
17. Cerebral Homeostasis.mp. [mp=ti, ab, hw, tn, ot, dm, mf, dv, kw, fx, dq, bt, id, cc, nm, kf, ox, px, rx, an, ui, sy]
18. cerebral auto regulation.mp. [mp=ti, ab, hw, tn, ot, dm, mf, dv, kw, fx, dq, bt, id, cc, nm, kf, ox, px, rx, an, ui, sy]
19. Intracranial Pressure.mp. [mp=ti, ab, hw, tn, ot, dm, mf, dv, kw, fx, dq, bt, id, cc, nm, kf, ox, px, rx, an, ui, sy]
20. Intracerebral Pressure.mp. [mp=ti, ab, hw, tn, ot, dm, mf, dv, kw, fx, dq, bt, id, cc, nm, kf, ox, px, rx, an, ui, sy]
21. Subarachnoid Pressure.mp. [mp=ti, ab, hw, tn, ot, dm, mf, dv, kw, fx, dq, bt, id, cc, nm, kf, ox, px, rx, an, ui, sy]
22. ICP.mp. [mp=ti, ab, hw, tn, ot, dm, mf, dv, kw, fx, dq, bt, id, cc, nm, kf, ox, px, rx, an, ui, sy]
23. Cerebral blood flow velocity.mp. [mp=ti, ab, hw, tn, ot, dm, mf, dv, kw, fx, dq, bt, id, cc, nm, kf, ox, px, rx, an, ui, sy]
24. 9 or 10 or 11 or 12 or 13 or 14 or 15 or 16 or 17 or 18 or 19 or 20 or 21 or 22 or 23
25. 8 and 24
26. remove duplicates from 25
